# The Clinicopathological and Prognostic Significance of Nrf2 and Keap1 Expression in Hepatocellular Carcinoma

**DOI:** 10.3390/cancers12082128

**Published:** 2020-07-31

**Authors:** Kiryang Lee, Seunghye Kim, Yangkyu Lee, Hyejung Lee, Youngeun Lee, Hyunjin Park, Ji Hae Nahm, Soomin Ahn, Su Jong Yu, Kyoungbun Lee, Haeryoung Kim

**Affiliations:** 1 Department of Pathology, Seoul National University Hospital, Seoul National University College of Medicine, Seoul 03080, Korea; pathologic2017@gmail.com (K.L.); pit_a_pat@snu.ac.kr (S.K.); kardia0622@gmail.com (H.L.); nayye@hanmail.net (Y.L.); azirang@gmail.com (K.L.); 2 Department of Pathology, Seoul National University Bundang Hospital, Seoul National University College of Medicine, Seongnam 13620, Korea; gyulgyuli@naver.com; 3 Department of Pathology, Gangnam Severance Hospital, Yonsei University College of Medicine, Seoul 06273, Korea; umberto0828@hotmail.co.kr (H.P.); nam2169@yuhs.ac (J.H.N.); 4 Department of Pathology and Translational Genomics, Samsung Medical Center, Sungkyunkwan University School of Medicine, Seoul 06351, Korea; suminy317@gmail.com; 5 Department of Internal Medicine and Liver Research Institute, Seoul National University College of Medicine; Biomedical Research Institute, Center for Medical Innovation, Seoul National University Hospital, Seoul 03080, Korea; ydoctor2@hanmail.net

**Keywords:** Hepatocellular carcinoma, Nrf2, Keap1, prognosis

## Abstract

Nuclear factor E2-related factor2 (Nrf2) activation is associated with both cytoprotective effects and malignant behavior of cancer cells. This study aimed to evaluate the clinicopathological implications of the expression of Nrf2, pNrf2, and its regulator Keap1 in human hepatocellular carcinomas (HCCs). Tissue microarrays consisting of 285 surgically resected HCCs were immunohistochemically stained with pNrf2, Nrf2, Keap1, stemness-related markers (keratin 19 (K19), epithelial cell adhesion molecule (EpCAM)), carbonic anhydrase IX (CAIX), epithelial–mesenchymal transition (EMT)-related markers (ezrin, uPAR, E-cadherin), and p53, and the results were correlated with the clinicopathological features. pNrf2 expression was significantly associated with increased proliferative activity, as well as EpCAM, ezrin, p53, and CAIX expression and E-cadherin loss (*p* < 0.05, all). Strong cytoplasmic Nrf2 expression was associated with CAIX and ezrin expression (*p* < 0.05, both). Keap1 was associated with increased proliferative activity, portal vein invasion, EMT-related markers, and p53 expression in CAIX-negative HCCs (*p* < 0.05, all). Both pNrf2 and cytoplasmic Nrf2 expression were associated with decreased overall survival (*p* < 0.05, both), and cytoplasmic Nrf2 expression was an independent predictor of decreased overall survival on multivariate analysis (hazard ratio 4.15, *p* < 0.001). Both pNrf2 and cytoplasmic Nrf2 expression were associated with poor survival and aggressive behavior of HCC. In addition, Keap1 expression was also associated with aggressive HCC behavior in CAIX-negative HCCs, suggesting that Keap1 expression should be interpreted in the context of hypoxia status.

## 1. Introduction

Hepatocellular carcinoma (HCC) is the fourth leading cause of cancer-related mortality worldwide [1]. The genomic landscape of HCC was unraveled during the past decade, with the most common genetic alterations being mutations in *TP53*, *CTNNB1*, and *TERT* promoter genes [2,3,4]; following the identification of distinct morphological subtypes of HCCs that partly correlate with the molecular features, there was increasing interest in the heterogeneity of HCC in recent years [5]. This heterogeneity has important clinical implications, as understanding the morphomolecular features of the different subtypes of HCC will lead to increased treatment options and provide background for drug development. 

Of the less frequent molecular alterations of HCC, somatic mutations in *NFE2L2* and *KEAP1* are observed in 3–6% and 2–8% of HCCs, respectively [4,6]. Activating *NFE2L2* mutations and inactivating *KEAP1* mutations were demonstrated to play important roles in tumor progression by preventing the degradation of nuclear factor E2-related factor2 (Nrf2), and the Nrf2/Keap1 pathway recently attracted attention as a potential therapeutic target for HCC [3,7,8]. Under physiological conditions, Nrf2 has cytoprotective functions; oxidative stress induces the phosphorylation of Nrf2, and phospho-Nrf2 (pNrf2) is subsequently released from Keap1 to enter the nucleus, bind to antioxidant response elements, and ultimately trigger the expression of cytoprotective genes [6,9,10]. However, although this cytoprotective mechanism initially prevents carcinogenesis, the overexpression of Nrf2 in cancer cells may result in protection of cancer cells from oxidative stress, enhancement of cancer cell proliferation and survival, and resistance to therapy [8,11,12,13,14]. 

Although the prognostic and clinical implication of Nrf2 and Keap1 protein expression was recently demonstrated in various malignant neoplasms, previous studies reported conflicting results and there are limited data on HCC [15,16,17,18,19,20,21,22]. In this study, we evaluated the prognostic significance and the clinicopathological features of HCCs expressing Nrf2, pNrf2, and Keap1, and we correlated the findings with the expression status of markers associated with hypoxia (carbonic anhydrase IX; CAIX), stemness (keratin 19; K19, epithelial cell adhesion molecule; EpCAM) and epithelial–mesenchymal transition (EMT) (ezrin, urokinase-type plasminogen activator receptor (uPAR) and E-cadherin).

## 2. Results

The clinicopathological features of the 285 HCC cases are summarized in Table 1. The most common etiology was hepatitis B virus (HBV) infection (203/285, 71.2%). Multiplicity was present in 49/285 (17.2%) cases, and 205/285 (72.0%) cases were poorly differentiated (Edmondson–Steiner grade 3 or 4). Microvascular and portal vein invasion was seen in 113/285 (39.6%) and 19/285 (6.7%) cases, respectively. After a follow up period of 66.5 months (range, 0–163 months), 161/285 (56.5%) cases recurred and 54/285 (18.9%) patients died. 

### 2.1. pNrf2 Expression in HCCs and Clinicopathological Correlation 

**Figure 1:**
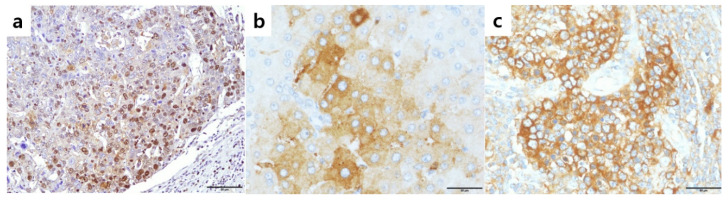
Expression of phosphorylated nuclear factor E2-related factor2 (pNrf2), Nrf2, and Keap1 in hepatocellular carcinomas. (**a**) Nuclear pNrf2 expression in 63% of tumor cells; (**b**) high cytoplasmic Nrf2 expression in tumor cells; (**c**) high cytoplasmic Keap1 expression in tumor cells. Original magnification ×200 (**a**), ×400 (**b**,**c**).

**Figure 2:**
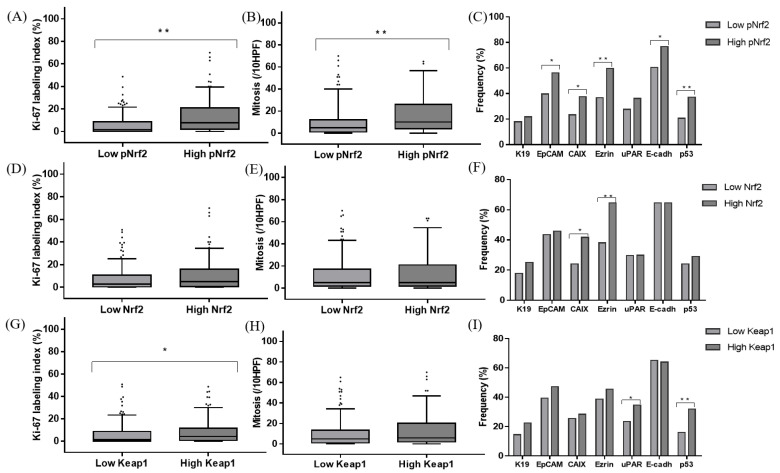
Comparison of the Ki-67 labeling indices (**A**,**D**,**G**), mitotic indices (**B**,**E**,**H**), and immunohistochemical characteristics (**C**,**F**,**I**) of hepatocellular carcinomas according to pNrf2 (**A**–**C**), Nrf2 (**D**–**F**), and Keap1 (**G**–**I**) expression status; * *p* < 0.05, ** *p* < 0.01; HPF: high-power field.

**Figure 3:**
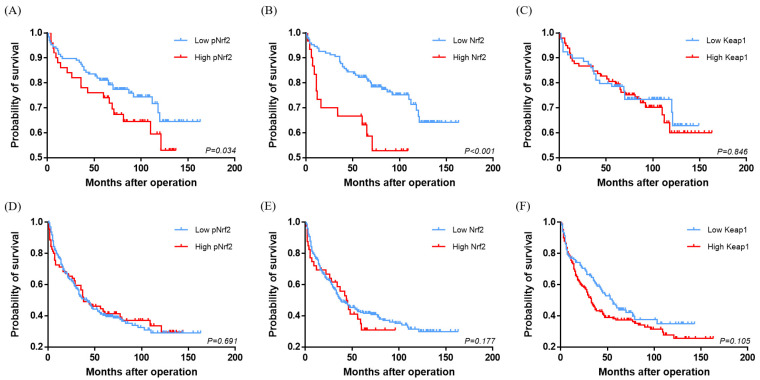
Kaplan–Meier survival curves comparing the overall survival (**A**–**C**) and disease-free survival (**D**–**F**) of hepatocellular carcinomas according to pNrf2 (**A**,**D**), Nrf2 (**B**,**E**), and Keap1 (**C**,**F**) expression status.

The immunohistochemical stain results and clinicopathological data are demonstrated in Figure 1 and Table 2. pNrf2 expression was observed in 73/285 (25.6%) cases, and all positive cases demonstrated strong nuclear labeling. High pNrf2 expression was associated with significantly higher proliferative activity (mitotic index: 17.4 ± 20.0% vs. 10.1 ± 13.4%, respectively, *p* < 0.001; Ki-67 labeling index (12.9 ± 12.2 vs. 5.1 ± 7.5/10 high-power field (HPF), respectively, *p* < 0.001) compared with pNrf2-low HCCs. pNrf2-high HCCs demonstrated significantly more frequent expression of EpCAM (41/73 (56.2%) vs. 84/212 (39.6%), respectively, *p* = 0.020), CAIX (27/72 (37.5%) vs. 48/205 (23.4%), respectively, *p* = 0.030), ezrin (43/72 (59.7%) vs. 78/212 (36.8%), respectively, *p* = 0.001), E-cadherin loss (56/73 (76.7%) vs. 128/212 (60.4%), respectively, *p* = 0.015), and p53 overexpression (26/70 (37.1%) vs. 40/195 (20.5%), respectively, *p* = 0.009), compared with pNrf2-low HCCs (Figure 2). K19 expression was also slightly more frequent in pNrf2-high HCCs; however, the difference was not statistically significant. pNrf2-high status was significantly associated with decreased overall survival (OS) on univariate analysis (hazard ratio (HR), 1.89: 95% confidence interval (CI), 1.04–3.46; *p* = 0.038) (Table 3, Figure 3). There was no significant difference in disease-free survival (DFS) according to pNrf2 status. However, analysis of an independent cohort of 293 surgically resected HCCs also revealed a significant association between pNrf2 expression and decreased OS (*p* = 0.001) and DFS (*p* = 0.029) (Figure S1).

### 2.2. Cytoplasmic Nrf2 Expression in HCCs and Clinicopathological Correlation 

Cytoplasmic Nrf2 expression was observed in 48/285 (16.8%) of HCCs. There was no correlation between pNrf2 and Nrf2 expression status. Nrf2-high HCCs were smaller in size compared with Nrf2-low HCCs (3.1 ± 1.8 cm vs. 4.4 ± 2.9 cm, respectively, *p* = 0.005). Cytoplasmic Nrf2 expression was associated with increased expression of CAIX (20/48 (41.7%) vs. 55/229 (24.0%), respectively, *p* = 0.019) and Ezrin (31/48 (64.6%) vs. 90/236 (38.1%), respectively, *p* = 0.001) compared to Nrf2-low HCCs. Notably, Nrf2-high status was significantly associated with decreased OS in HCC patients on univariate analysis (HR, 3.33; 95% CI, 1.74–6.37; *p* < 0.001) (Table 3, Figure 3).

### 2.3. Keap1 Expression in HCCs and Clinicopathological Correlation

Keap1 was expressed in the cytoplasm of 161/285 (56.5%) HCCs. Interestingly, a significant positive correlation was observed between pNrf2 and Keap1 expression (*p* = 0.020). Keap1 expression was associated with increased expression of uPAR (29/124 (23.4%) vs. 55/159 (34.6%), respectively, *p* = 0.049). In addition, more frequent p53 expression (47/147 (32.0%) vs. 19/118 (16.1%), respectively, *p* = 0.004) and higher proliferative activity (Ki-67 labeling index: 8.1 ± 10.2% vs. 5.7 ± 8.5%, respectively, *p* = 0.035; mitotic index: 13.1 ± 16.9/10 HPF vs. 10.5 ± 13.6/10 HPF, respectively, *p* = 0.056) were noted in Keap1-high HCCs, compared to Keap1-low HCCs.

The positive correlation between pNrf2 and Keap1 and the association between Keap1 and increased proliferative activity in this study were unexpected, as Keap1 is known to suppress Nrf2 activation and, thus, expected to have tumor-suppressive effects. We, thus, analyzed the clinicopathological features of Keap1-positive HCCs separately in CAIX-positive (*n* = 75) and CAIX-negative HCCs (*n* = 202). Of note, we found that Keap1 expression was associated with increased Ki-67 labeling index (7.4 ± 10.3% vs. 4.6 ± 7.1%, respectively, *p* = 0.008), more frequent portal vein invasion (1/202 (0.9%) vs. 6/75 (13.6%), respectively, *p* = 0.024), ezrin expression (3/91 (3.3%) vs. 13/110 (11.8%), respectively, *p* = 0.035), uPAR expression (15/91 (16.5%) vs. 33/110 (30.0%), respectively, *p* = 0.031), and p53 expression (31/202 (29.8%) vs. 14/75 (34.1%), respectively, *p* = 0.009) only in CAIX-negative HCCs, while there was no association between Keap1 status and the clinicopathological factors in CAIX-positive HCCs (Table S1). In addition, although there were no differences in patient survival according to Keap1 expression status, there was interestingly a tendency for decreased DFS for Keap1-high HCCs in the CAIX-negative HCC group (*p* = 0.062).

### 2.4. Multivariate Survival Analysis

Multivariate survival analysis was performed with clinicopathological and immunohistochemical parameters that demonstrated statistical significance on univariate analysis, including pNrf2 and cytoplasmic Nrf2. Other parameters associated with decreased OS on univariate analysis included tumor multiplicity (HR, 2.81; 95% CI, 1.49–5.31; *p* = 0.001), tumor size (≥5 cm vs. <5 cm) (HR, 2.40; 95% CI, 1.32–4.38; *p* = 0.004), portal vein invasion (HR, 3.29; 95% CI, 1.39–7.80; *p* = 0.007), the presence of extrahepatic metastasis (HR, 4.46; 95% CI, 2.45–8.13; *p* < 0.001), and expression of CAIX (HR, 1.85; 95% CI, 1.02–3.39; *p* = 0.044) and ezrin (HR, 3.16; 95% CI, 1.67–5.97; *p* < 0.001). Multivariate analysis revealed cytoplasmic Nrf2 expression to be a significant independent prognostic factor for OS (HR, 4.35; 95% CI, 2.11–8.97; *p* < 0.001), along with tumor multiplicity (HR, 1.98; 95% CI, 1.02–3.86; *p* = 0.045), presence of extrahepatic metastasis (HR, 3.75; 95% CI, 1.91–7.36; *p* < 0.001), and ezrin expression (HR, 2.41; 95% CI, 1.25–4.64; *p* = 0.009) (Table 3). As for DFS, tumor multiplicity (HR, 1.49; 95% CI, 1.02–2.18; *p* = 0.042) and the presence of extrahepatic metastasis (HR, 4.48; 95% CI, 3.15–6.35; *p* < 0.001) were the only significant independent predictors on multivariate analysis, and none of the immunohistochemical markers were associated with poor DFS.

## 3. Discussion

In this study, we examined the expression of Nrf2, pNrf2, and Keap1 in HCCs, and we correlated the findings with the clinicopathological parameters and expression status of stemness, hypoxia, and EMT-related markers. The association between Nrf2 expression and poor prognosis was previously demonstrated in various solid malignancies, including in two meta-analysis studies [18,21]; however, the prognostic value of Nrf2/Keap1 in HCCs was only reported in two studies so far [16,22]. Zhang et al. demonstrated an association between Nrf2 expression in HCCs and decreased survival, poor histological differentiation, increased proliferation, and more frequent metastasis [22]. Although we observed a significant association between strong cytoplasmic Nrf2 expression in HCC with decreased OS and EMT-related marker expression, other clinicopathological factors such as proliferative activity were not significantly influenced by Nrf2 expression status. The different antibodies used may partly explain the differences; Zhang et al. described nucleocytoplasmic Nrf2 expression with predominantly nuclear localization in their study, while Nrf2 expression in the current study was cytoplasmic. As it is the nuclear form of Nrf2 that is recognized to be the active pro-tumorigenic form, we also examined nuclear pNrf2 expression in HCC, and we found that pNrf2-high HCCs were associated with poor prognosis, increased proliferative activity, and more frequent expression of markers associated with stemness, EMT, and hypoxia. The association between pNrf2 expression and decreased survival in HCCs is in line with a previous study by Chen et al., although we did not find the same expected positive correlation between pNrf2 and Nrf2 expression or an inverse correlation between pNrf2 and Keap1 expression that they observed [16]. It should be noted that Chen et al. reported a larger proportion of pNrf2-positive HCCs (50%) which could be explained by the different cut-offs; we evaluated pNrf2 using a quantitative approach and defined “pNrf2-high” HCCs (~25%) as those with strong nuclear labeling exceeding 10%, while the former study adopted a semiquantitative approach for both pNrf2 and Keap1, counting any labeling for pNrf2 and Keap1 (in the nucleus and cytoplasm, respectively) that was more than focal weak positive as “positive” [16]. Nevertheless, despite the differences in interpretation methods, it is interesting that pNrf2 expression was associated with CAIX, EpCAM, K19, and ezrin expression in this study.

A subset of HCCs that are morphologically compatible with HCC but express immunohistochemical markers associated with stemness (e.g., K19, EpCAM, and SAL-like protein 4 (SALL4)) recently received increasing attention, as these HCCs demonstrate more aggressive behavior compared to conventional HCCs, expression of EMT-related markers, poor prognosis, and resistance to locoregional and systemic treatment [23,24,25]. HCCs expressing stemness-related markers were shown to more frequently result in residual tumors after transarterial chemoembolization (TACE), compared to conventional HCCs, and the residual tumors after TACE often expressed stemness-related markers that were co-localized with CAIX expression, thus suggesting a relationship between TACE-induced hypoxia and stemness [26]. In this study, we found positive correlations between pNrf2 expression and hypoxia, stemness, and EMT-related markers, suggesting a possible role for Nrf2 in the link between tumor hypoxia, stemness, and aggressive behavior of HCC. pNrf2 may be upregulated in HCCs in response to oxidative stress, and pNrf2 may further enhance the aggressive behavior of hypoxic HCCs; however, functional studies would be necessary for validation.

Although Keap1 expression status was not correlated with clinicopathological factors or patient survival, Keap1-high HCCs more frequently demonstrated p53 expression and higher proliferative activity. In addition, a positive correlation was found between Keap1 and pNrf2 expression status. These results are somewhat counterintuitive, as Keap1 was described to be a protein that suppresses Nrf2 activation. Interestingly, when we examined the transcriptomics data provided by The Human Protein Atlas (https://www.proteinatlas.org/) and UALCAN (http://ualcan.path.uab.edu/), which are based on The Cancer Genome Atlas (TCGA) data, we found that *KEAP1* messenger RNA (mRNA) expression was in fact higher in HCCs than in non-neoplastic liver tissues, and high *KEAP1* mRNA was associated with poor prognosis in HCCs (Figure S2) [27,28]. An association between Keap1 expression and poor prognosis was also demonstrated in endometrial cancers, and the authors postulated that the overexpression of Keap1 may reflect Keap1 induction in the setting of oxidative stress [15]. On the other hand, when we analyzed the Keap1 expression status separately in CAIX-positive and CAIX-negative HCCs, we interestingly found that Keap1 expression was associated with more frequent portal vein invasion, higher proliferative activity, frequent p53 expression, and a tendency for decreased DFS in CAIX-negative HCCs, while Keap1 was not associated with any of the clinicopathological or immunohistochemical factors in the CAIX-positive HCCs. Instead, Nrf2 and/or pNrf2 were the determinants of aggressive behavior and poor survival in the setting of CAIX-positive HCCs. Thus, it is possible that, under hypoxic stress, Nrf2 is released from Keap1 to contribute to the aggressive behavior of HCC, while, under normoxic conditions, Keap1 is also a determinant of aggressive behavior. In addition, the association between strong cytoplasmic Nrf2 expression and decreased OS in this study suggests that, although the nuclear pNrf2 is the active form of Nrf2, strong overexpression of Nrf2 in the cytoplasm is also abnormal and has prognostic implications.

There are a few limitations to this study. Firstly, although we analyzed a large number of HCCs, this is a retrospective cohort study using formalin-fixed paraffin-embedded tissue samples and the results are based on immunohistochemical results. As all the HCCs enrolled in this study were surgically resected HCCs, it is possible that early well-differentiated HCCs—which are often treated by loco-regional modalities—were less likely to be included in this study, resulting in a biased cohort predominantly consisting of moderately to poorly differentiated HCCs. Functional studies would need to be performed to further evaluate the functional relationships among Nrf2/Keap1, hypoxia, and EMT. Secondly, as the majority of the HCCs in this study have an HBV-related etiology, it would be necessary to perform a similar analysis on an independent cohort of HCCs from regions where HBV is not as predominant.

## 4. Materials and Methods

### 4.1. Patients

A total of 285 cases of primary HCCs that were surgically resected between 2003 and 2012 at Seoul National University Bundang Hospital (Seongnam, Korea) were evaluated in this study. Clinicopathological information was obtained from the patients’ electronic medical records, pathology database, and a review of the archived glass slides, and it included sex, age at operation, underlying etiology (including hepatitis B virus (HBV), hepatitis C virus (HCV), alcohol and non-alcoholic fatty liver disease (NAFLD)), tumor size, multiplicity, tumor differentiation (Edmonson–Steiner grade), presence of microvascular and portal vein invasion, mitotic index, extrahepatic metastasis, and pathological T stages according to the American Joint Committee on Cancer TNM staging system (eighth edition). The mitotic index was expressed as the total number of mitotic figures in 10 high-power fields (HPFs). The clinical follow-up information, including the dates of surgery, disease recurrence, death, and most recent follow-up, was retrieved from the electronic medical records. Overall survival (OS) was defined as the number of months between surgery and patient death, and disease-free survival (DFS) was defined as the number of months between surgery and disease recurrence (local recurrence and/or distant metastasis). In addition, a separate independent tissue microarray cohort of 293 resected HCCs from Seoul National University Hospital was evaluated to further examine the prognostic significance of pNrf2 expression. This study was approved by the Institutional Review Board of Seoul National University Hospital and Seoul National University Bundang Hospital (joint IRB# H-1808-073-965), and patient consent was waived due to the retrospective nature of this study.

### 4.2. Tissue Samples and Immunohistochemistry

Tissue microarrays with tissue cores measuring 2 mm in diameter were constructed from formalin-fixed paraffin-embedded HCC tissue, and they were arranged in recipient tissue microarray blocks using a trephine apparatus (Superbiochips Laboratories, Seoul, Korea). Immunohistochemistry was performed on 4-μm-thick tissue sections using antibodies for pNrf2, Nrf2, Keap1, K19, EpCAM, E-cadherin, CAIX, p53, and Ki-67, using an automated platform (Ventana BenchMark GX, Ventana Medical Systems, Oro Valley, AZ, USA). Immunohistochemical staining for ezrin and uPAR was performed manually, with antigen retrieval using the citrate buffer (pH 6.0) and the Envision Kit (Dako, Glostrup, Denmark). Detailed information on the antibodies is summarized in Table 4.

For Nrf2 and Keap1, the intensity of cytoplasmic expression in the tumor cells was recorded as 0 (negative), 1+ (weak), 2+ (moderate), and 3+ (strong). Nrf2 expression was cytoplasmic, and strong positive (3+) staining was designated as “Nrf2-high” (overexpression). “Keap1-high” was defined as moderate to strong cytoplasmic staining in the tumor cells, and the staining intensity was similar to or more intense than that seen in non-neoplastic hepatocytes, cholangiocytes, and stromal cells. pNrf2 staining was evaluated in a quantitative manner, using the ImageJ software (National Institutes of Health, Bethesda, MD, USA); the percentage of nuclear pNrf2 labeling was calculated by dividing the number of positively stained tumor cell nuclei by the total number of tumor cell nuclei, in an HPF (400× magnification). “pNrf2-high” was defined as pNrf2 labeling in ≥10% of tumor cells. K19, EpCAM, CAIX, ezrin, and uPAR expression was designated positive when there was cytoplasmic (K19, CAIX, ezrin) or membranous (EpCAM and uPAR) expression in ≥5% of the tumor cells. Loss of E-cadherin expression was defined as complete absence of membranous E-cadherin expression in the tumor cells. p53 expression was defined as positive when strong nuclear labeling was present in more than 10% HCC cells. Two pathologists (K.L. and H.K.) independently evaluated the immunohistochemical stains and reached an agreement using a multihead microscope when there were discrepancies in the interpretation results. 

### 4.3. Statistical Analysis

All data were analyzed using IBM SPSS statistics software version 25.0 (SPSS Inc., Chicago, IL, USA) and GraphPad Prism software version 7.0 (San Diego, CA, USA), and a *p*-value <0.05 was considered statistically significant. The associations between the immunohistochemical stain results and clinicopathological parameters were assessed using the χ^2^ test, Fisher exact test, and Student’s *t*-test, as deemed appropriate. OS and DFS were analyzed using the Kaplan–Meier method and the log-rank test. Multivariate analysis using the Cox regression model was performed for clinicopathological and immunohistochemical parameters with *p* < 0.05 on univariate analysis. 

## 5. Conclusions

In summary, we demonstrate that pNrf2 expression in HCC was associated with increased proliferative activity, decreased survival, and more frequent expression of hypoxia, stemness, and EMT-related markers, suggesting a possible role for pNrf2 as a marker of aggressive behavior and poor prognosis in HCC. In addition, contrary to previous knowledge, strong cytoplasmic Nrf2 expression was also an independent predictor of decreased survival in HCC, and Keap1 expression was associated with aggressive HCC behavior in CAIX-negative tumors, suggesting that Keap1 expression in HCCs should be interpreted in the context of the metabolic status. Further prognostic studies in independent cohorts and functional studies would be required to validate the current findings.

## Figures and Tables

**Table 1:** Summary of the clinicopathological features of hepatocellular carcinomas (*n* = 285).

Parameters	Frequency (%) or Median (Range)
Sex, *n* (%)	
Male	216 (75.8)
Female	69 (24.2)
Age at operation, median (range, years)	63 (29–87)
Etiology, *n* (%)	
Hepatitis B virus (HBV)	202 (70.9)
Hepatitis C virus (HCV)	25 (8.8)
HBV + HCV	1 (0.3)
Alcohol	14 (4.9)
Non-alcoholic fatty liver disease	12 (4.2)
Uncertain etiology	31 (10.9)
Tumor size, median (range, cm)	3.2 (0.9–17)
Multiplicity present, *n* (%)	49 (17.2)
Edmondson–Steiner grade, *n* (%)	
Grade I	3 (1.0)
Grade II	77 (27.0)
Grade III	171 (60.0)
Grade IV	34 (12.0)
Microvascular invasion present, *n* (%)	113 (39.6)
Portal vein invasion present, *n* (%)	19 (6.7)
Pathologic T category (AJCC 8th edition), *n* (%)	
pT1a	37 (13.0)
pT1b	101 (35.4)
pT2	112 (39.3)
pT3	11 (3.9)
pT4	24 (8.4)
Extrahepatic metastasis present, *n* (%)	55 (19.3)
Cirrhosis in background liver present, *n* (%)	152 (533.3)
Recurrence on follow-up present, *n* (%)	161 (56.5)
Status at last follow-up, *n* (%)	
Alive	128 (44.9)
Deceased of disease	44 (15.4)
Deceased of other cause	10 (3.5)
Follow up loss	103 (36.2)
Overall survival, median (range, months)	66.5 (0–163)
Disease-free survival, median (range, months)	32.5 (0–163)

AJCC, American Joint Committee on Cancer.

**Table 2:** Summary of the clinicopathological and immunohistochemical features of hepatocellular carcinomas according to pNrf2, Nrf2 and Keap1 status (*n* = 285), *n* (%).

Parameter	pNrf2		*p*-Value	Nrf2		*p*-Value	Keap1		*p*-Value
	Low pNrf2	High pNrf2		Low Nrf2	High Nrf2		Low Keap1	High Keap1	
Frequency	212 (74.3)	73 (25.6)	-	237 (83.2)	48 (16.8)	-	124 (43.5)	161 (56.5)	-
Sex (male/female)	159 (75.0)/53 (25.0)	57 (78.1)/16 (21.9)	0.638	179 (75.5)/58 (24.5)	37 (77.1)/11 (22.9)	1.000	94 (75.8)/30 (24.2)	122 (75.8)/39 (24.2)	1.000
Age ≥60 years	95 (44.8)	28 (38.4)	0.411	100 (42.2)	23 (47.9)	0.542	55 (44.7)	68 (42.2)	0.809
HBV-related etiology	150 (70.8)	53 (72.6)	0.881	167 (70.5)	36 (75.0)	0.602	86 (69.4)	117 (72.7)	0.598
Size (cm, mean ± SD)	4.2 ± 3.0	4.0 ± 2.4	0.274	4.4 ± 2.9	3.1 ± 1.8	0.005	4.4 ± 3.1	3.9 ± 2.5	0.105
Multiplicity	37 (17.5)	12 (16.4)	1.000	41 (17.3)	8 (16.7)	1.000	23 (18.5)	26 (16.1)	0.636
E–S grade III and IV	155 (73.1)	50 (68.5)	0.454	173 (73.0)	32 (66.7)	0.382	92 (74.2)	113 (70.2)	0.507
Microvascular invasion	78 (36.8)	35 (47.9)	0.098	96 (40.5)	17 (35.4)	0.628	47 (37.9)	66 (41.0)	0.627
Portal vein invasion	12 (5.7)	7 (9.6)	0.278	17 (7.2)	2 (4.2)	0.750	12 (9.7)	7 (4.3)	0.094
pT category			0.146			0.323			0.166
pT1a	31 (14.6)	6 (8.2)		33 (13.9)	4 (8.3)		18 (14.5)	19 (11.8)	
pT1b	75 (35.4)	26 (35.6)		83 (35.0)	18 (37.5)		46 (37.1)	55 (34.2)	
pT2	76 (35.8)	36 (49.3)		89 (37.6)	23 (47.9)		41 (33.1)	71 (44.1)	
pT3	10 (4.7)	1 (1.4)		11 (4.6)	0 (0)		4 (3.2)	7 (4.3)	
pT4	20 (9.4)	4 (5.5)		21 (8.9)	3 (6.3)		15 (12.1)	9 (5.6)	
Recurrence	118 (55.7)	43 (58.9)	0.784	133 (56.1)	28 (58.3)	0.748	64 (51.6)	97 (60.2)	0.183
Extrahepatic metastasis	37 (17.6)	18 (24.7)	0.229	50 (21.2)	5 (9.1)	0.109	24 (19.5)	31 (19.4)	1.000
Underlying cirrhosis	108 (52.9)	44 (60.3)	0.338	125 (54.1)	27 (58.7)	0.628	65 (55.1)	87 (54.7)	1.000
K19 expression	38 (17.9)	16 (21.9)	0.490	42 (17.7)	12 (25.0)	0.233	18 (14.5)	36 (22.4)	0.127
EpCAM expression	84 (39.6)	41 (56.2)	0.020	103 (43.5)	22 (45.8)	0.873	49 (39.5)	76 (47.2)	0.229
CAIX expression	48 (23.4)	27 (37.5)	0.030	55 (24.0)	20 (41.7)	0.019	31 (25.4)	44 (28.4)	0.589
Ezrin expression	78 (36.8)	43 (59.7)	0.001	90 (38.1)	31 (64.6)	0.001	48 (38.7)	73 (45.6)	0.277
uPAR expression	58 (27.5)	26 (36.1)	0.180	70 (29.7)	14 (29.8)	1.000	29 (23.4)	55 (34.6)	0.049
E-cadherin loss	128 (60.4)	56 (76.7)	0.015	153 (64.6)	31 (64.6)	1.000	81 (65.3)	103 (64.0)	0.901
p53 overexpression	40 (20.5)	26 (37.1)	0.009	53 (24.1)	13 (28.9)	0.570	19 (16.1)	47 (32.0)	0.004
Mitotic index (/10 HPF, mean ± SD)	10.1 ± 13.4	17.4 ± 20.0	<0.001	11.9 ± 15.6	12.2 ± 16.1	0.623	10.5 ± 13.6	13.1 ± 16.9	0.056
Ki-67 labeling index (%, mean ± SD)	5.1 ± 7.5	12.9 ± 12.2	<0.001	7.2 ± 9.6	6.7 ± 9.6	0.935	5.7 ± 8.5	8.1 ± 10.2	0.035

HR: hazard ratio, CI: confidence interval, HBV: hepatitis B virus, E–S grade: Edmondson–Steiner grade, K19: keratin 19, EpCAM: epithelial cell adhesion molecule, CAIX: carbonic anhydrase IX; uPAR: urokinase-type plasminogen activator receptor, HPF: high-power field.

**Table 3:** Summary of the survival analysis results (*n* = 285).

Parameter	Overall Survival				Disease-Free Survival			
	Univariate Analysis		Multivariate Analysis		Univariate Analysis		Multivariate Analysis	
Parameters	HR (95% CI)	*p*-Value	HR (95% CI)	*p*-Value	HR (95% CI)	*p*-Value	HR (95% CI)	*p*-Value
Sex (male/female)	0.742 (0.345–1.597)	0.446	-	-	0.702 (0.480–1.029)	0.069	-	-
Age (≥60/<60 years)	0.960 (0.522–1.765)	0.894	-	-	0.848 (0.621–1.157)	0.299	-	-
Underlying HBV	1.160 (0.586–2.296)	0.671	-	-	1.129 (0.801–1.590)	0.488	-	-
Size (≥5cm/<5cm)	2.400 (1.315–4.382)	0.004	1.298 (0.650–2.594)	0.460	1.418 (1.012–1.988)	0.043	1.085 (0.765–1.540)	0.647
Multiplicity	2.812 (1.488–5.312)	0.001	2.156 (1.093–4.253)	0.027	1.991 (1.375–2.884)	<0.001	1.487 (1.015–2.180)	0.042
E–S grade III and IV	0.951 (0.497–1.818)	0.879	-	-	1.095 (0.778–1.542)	0.601	-	-
Microvascular invasion	1.477 (0.813–2.682)	0.200	-	-	1.308 (0.962–1.779)	0.087	-	-
Portal vein invasion	3.289 (1.386–7.804)	0.007	2.435 (0.980–6.052)	0.055	1.573 (0.853–2.903)	0.147	-	-
Extrahepatic metastasis	4.458 (2.445–8.130)	<0.001	3.256 (1.742–6.083)	<0.001	4.799 (3.411–6.753)	<0.001	4.475 (3.152–6.354)	<0.001
Underlying cirrhosis	1.282 (0.702–2.340)	0.419	-	-	1.031 (0.758–1.404)	0.844	-	-
K19	1.860 (0.957–3.614)	0.067	-	-	1.209 (0.826–1.771)	0.329	-	-
EpCAM	1.727 (0.951-3.137)	0.073	-	-	0.889 (0.613–1.292)	0.538	-	-
CAIX	1.854 (1.015–3.386)	0.044	1.402 (0.738–2.664)	0.302	1.043 (0.743–1.464)	0.809	-	-
Ezrin	3.160 (1.674–5.966)	<0.001	2.893 (1.527–5.483)	0.001	1.090 (0.766–1.552)	0.632	-	-
uPAR	1.492 (0.812–2.743)	0.198			1.058 (0.756–1.480)	0.742		
E-cadherin loss	1.037 (0.556–1.936)	0.909			0.853 (0.622–1.169)	0.323		
p53 overexpression	1.777 (0.961–3.288)	0.067	-	-	1.219 (0.854–1.740)	0.276	-	-
Nrf2	3.329 (1.738–6.374)	<0.001	4.151 (2.025–8.508)	<0.001	1.315 (0.879–1.968)	0.183	-	-
pNrf2	1.894 (1.037–3.458)	0.038	1.181 (0.950–3.450)	0.071	0.933 (0.661–1.317)	0.693	-	-
Keap1	1.061 (0.582–1.937)	0.846	-	-	1.290 (0.944–1.762)	0.109	-	-

HBV: hepatitis B virus, HR: hazard ratio, CI: confidence interval, SD: standard deviation, E–S grade: Edmondson–Steiner grade, K19: keratin 19, EpCAM: epithelial cell adhesion molecule, CAIX: carbonic anhydrase IX, uPAR: urokinase-type plasminogen activator receptor, HPF: high-power field.

**Table 4:** Summary of the antibodies used in this study.

Antibody	Description	Source	Dilution	Method
Nrf2	Rabbit monoclonal (EP1808Y)	Abcam	1:50	Autostainer
		(Cambridge, UK)		
pNrf2	Rabbit monoclonal (EP1809Y)	Abcam	1:100	Autostainer
		(Cambridge, UK)		
Keap1	Rabbit polyclonal	Proteintech	1:300	Autostainer
		(Manchester, UK)		
Keratin 19 (K19)	Mouse monoclonal (RCK108)	Dako	1:200	Autostainer
		(Glostrup, Denmark)		
EpCAM	Mouse monoclonal (VU-1D9)	Millipore	1:1500	Autostainer
		(Temecula, CA, USA)		
Ezrin	Mouse monoclonal (3C12)	Abcam	1:100	Manual (pH 6.0)
		(Cambridge, UK)		
uPAR	Mouse monoclonal (R-4)	Abcam	1:40	Manual (pH 6.0)
		(Cambridge, UK)		
E-cadherin	Mouse monoclonal (36B5)	Novocastra	1:100	Autostainer
		(Newcastle, UK)		
CAIX	Rabbit polyclonal	Abcam	1:500	Autostainer
		(Cambridge, UK)		
p53	Mouse monoclonal (DO-7)	Dako	1:1000	Autostainer
		(Glostrup, Denmark)		
Ki-67	Mouse monoclonal (MIB-1)	Dako	1:100	Autostainer
		(Glostrup, Denmark)		

## Supplementary Materials

The following are available online at https://www.mdpi.com/2072-6694/12/8/2128/s1. Figure S1: Kaplan-Meier survival curves demonstrating decreased overall survival (A) and disease-free survival (B) in pNrf2-high HCCs in an independent cohort of HCC (*n* = 293), Figure S2: Increased KEAP1 mRNA expression in HCCs compared to non-neoplastic liver tissue (A) and decreased overall survival in KEAP1-high HCCs (B) in the LIHC cohort from The Cancer Genome Atlas (graphs generated from UALCAN, http://ualcan.path.uab.edu/); Table S1. Clinicopathologic features according to Keap1 expression status in CAIX-positive and CAIX-negative hepatocellular carcinomas.
