# Supplementary material for: The Clinicopathological and Prognostic Significance of Nrf2 and Keap1 Expression in Hepatocellular Carcinoma
Source: Cancers (Basel). 2020 Jul 31;12(8):2128. doi: 10.3390/cancers12082128 (PMC7464028; doi:10.3390/cancers12082128)
Supplement: Supplementary file 1 [file cancers-12-02128-s001.pdf]

Supplementary Files

The Clinicopathological and Prognostic Significance of Nrf2 and Keap1 Expression in Hepatocellular Carcinoma

Kiryang Lee, Seunghye Kim, Yangkyu Lee, Hyejung Lee, Youngeun Lee, Hyunjin Park, Ji Hae Nahm, Soomin Ahn, Su Jong Yu, Kyoungbun Lee and Haeryoung Kim

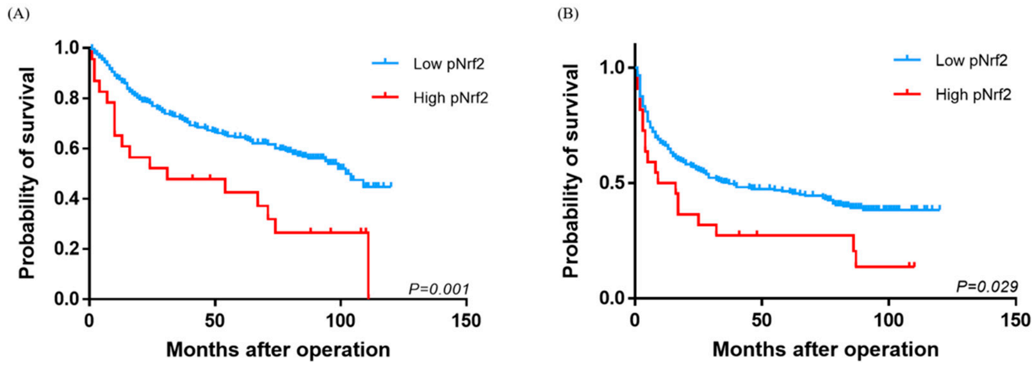

Figure S1. Kaplan-Meier survival curves demonstrating decreased overall survival (**A**) and disease-free survival (**B**) in pNrf2-high HCCs in an independent cohort of HCC (n = 293).

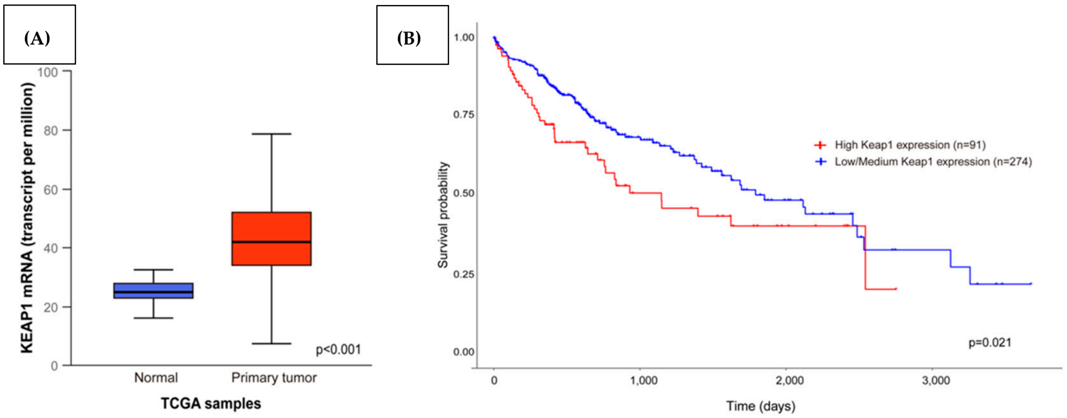

Figure S2. Increased KEAP1 mRNA expression in HCCs compared to non-neoplastic liver tissue (**A**) and decreased overall survival in KEAP1-high HCCs (**B**) in the LIHC cohort from The Cancer Genome Atlas (graphs generated from UALCAN, http://ualcan.path.uab.edu/).

Table S1. Clinicopathologic features according to Keap1 expression status in CAIX-positive and CAIX-negative hepatocellular carcinomas.

	**CAIX-negative (n=202)**		***P-value***	**CAIX-Positive (*n* = 75)**		***P-Value***
	**Low Keap1 (%)**	**High Keap1 (%)**		**Low Keap1 (%)**	**High Keap1 (%)**	
Frequency	174 (86.1)	28 (13.9)		55 (73.3)	20 (26.7)	
Sex (M/F)	68 (74.7)/ 23 (25.3)	85 (76.6)/ 26 (23.4)	0.869	25 (80.6)/ 6 (19.4)	32 (72.7)/ 12 (27.3)	0.584
Age ≥ 60 years	41 (45.1)	44 (39.6)	0.476	13 (41.9)	20 (45.5)	0.816
Underlying HBV	59 (64.8)	86 (77.5)	**0.059**	25 (80.6)	27 (61.4)	0.083
Size (cm, mean±SD)	4.0±2.8	3.8±2.6	0.955	5.8±3.6	4.3±2.5	**0.008**
Multiplicity	15 (16.5)	15 (13.5)	0.559	7 (22.6)	10 (22.7)	1.000
E-S grade III & IV	64 (70.3)	74 (66.7)	0.649	27 (87.1)	35 (79.5)	0.539
Microvascular invasion	35 (38.5)	39 (35.1)	0.661	12 (38.7)	24 (54.5)	0.241
Portal vein invasion	7 (7.7)	1 (0.9)	**0.024**	5 (16.1)	6 (13.6)	0.754
T stage			0.508			0.568
T1a	15 (16.5)	11 (9.9)		3 (9.7)	7 (15.9)	
T1b	35 (38.5)	43 (38.7)		10 (32.3)	11 (25.0)	
T2	31 (34.1)	48 (43.2)		10 (32.3)	19 (43.2)	
T3	3 (3.3)	5 (4.5)		1 (3.2)	2 (4.5)	
T4	7 (7.7)	4 (3.6)		7 (22.6)	5 (11.4)	
Extrahepatic metastasis	18 (20.0)	23 (20.9)	1.000	6 (19.4)	8 (18.2)	1.000
Underlying cirrhosis	45 (52.3)	58 (53.2)	1.000	18 (60.0)	27 (61.4)	1.000
K19 expression	10 (11.0)	20 (18.0)	0.171	8 (25.8)	15 (34.1)	0.612
EpCAM expression	14 (15.4)	25 (22.5)	0.215	14 (45.2)	29 (65.9)	0.098
Ezrin expression	3 (3.3)	13 (11.8)	**0.035**	17 (54.8)	29 (65.9)	0.348
uPAR expression	15 (16.5)	33 (30.0)	**0.031**	14 (45.2)	21 (48.8)	0.816
E-cadherin loss	55 (60.4)	69 (62.2)	0.885	25 (80.6)	32 (72.7)	0.584
p53 overexpression	12 (13.5)	31 (29.8)	**0.009**	7 (24.1)	14 (34.1)	0.434
Mitotic index (/10 HPF, mean±SD)	8.4±11.3	10.4±15.8	0.097	16.1±17.6	19.9±18.5	0.476
Ki-67 labeling index (%, mean±SD)	4.6±7.1	7.4±10.3	**0.008**	9.1±11.4	10.5±10.2	0.819

HBV: hepatitis B virus, SD: standard deviation, E-S grade: Edmondson-Steiner grade, K19: keratin 19, EpCAM: epithelial cell adhesion molecule, CAIX: carbonic anhydrase IX, HPF: high-power field.

© 2020 by the authors. Licensee MDPI, Basel, Switzerland. This article is an open access article distributed under the terms and conditions of the Creative Commons Attribution (CC BY) license (http://creativecommons.org/licenses/by/4.0/).
